# Impact of COVID-19 on Swimming Training: Practical Recommendations during Home Confinement/Isolation

**DOI:** 10.3390/ijerph18094767

**Published:** 2021-04-29

**Authors:** Monoem Haddad, Zied Abbes, Iñigo Mujika, Karim Chamari

**Affiliations:** 1 Physical Education Department, College of Education, Qatar University, Doha 2713, Qatar; ziedabbes@live.fr; 2 Department of Physiology, Faculty of Medicine and Nursing, University of the Basque Country, Basque Country, 48940 Leioa, Spain; inigo.mujika@inigomujika.com; 3 Exercise Science Laboratory, School of Kinesiology, Faculty of Medicine, Universidad Finis Terrae, Santiago 7501015, Chile; 4 ASPETAR, Qatar Orthopaedic and Sports Medicine Hospital, Doha 29222, Qatar; karim.chamari@aspetar.com; 5 Laboratory “Sport Performance Optimization”, (CNMSS), ISSEP Ksar-Said, Manouba University, Manouba 2010, Tunisia

**Keywords:** COVID-19, swimming, confinement, detraining, dryland, training methods

## Abstract

The COVID-19 pandemic has had severe effects on communities globally, leading to significant restrictions on all aspects of society, including in sports. Several significant decisions were made to postpone or cancel major swimming events by FINA (Fédération Internationale de Natation). Swimmers were no longer allowed to continue their usual training in swimming pools and were confined to their homes. These unusual circumstances may represent a good opportunity to strengthen different areas of swimmer preparation and potentially enhance performance when resuming regular aquatic training. We searched major databases for relevant information, and the present article provides practical information on home-based training for swimmers of all ages. The COVID-19 crisis and its consequences on the swimming community have created a myriad of challenges for swimmers around the world, including maintaining their fitness level and preparing to return optimally and safely to pool training and competitions. Unfortunately, the mental consequences that might arise after the pandemic may also have an impact. We strongly recommend encouraging the swimmers to consider quarantine as an opportunity for development in specific areas of preparation and learn how to best cope with this special situation of self-isolation and/or “physical distancing” for their mental health and in case a similar situation is faced again in the future.

## 1. Introduction

The coronavirus disease 2019 (COVID-19) caused by the severe acute respiratory syndrome coronavirus 2 (SARS-CoV-2) has had a severe effect on different communities around the word [1], leading to many restrictions on multiple areas of society, including sports. Many governments requested their population to stay home except for urgent or necessary outings [2]. Swimmers around the world have found themselves in a unique situation in which they were not only obliged to stop their usual athletic activity in swimming pools but were also confined to their homes.

Multiple features in swimming vary completely from other kind of sports, for example: the horizontal body position in the water, the forward propulsion with both arms and both legs at the same time (butterfly and breaststroke) [3], the necessity of the coordination between breathing patterns, buoyancy, and stroke efficiency [4], a higher tidal volume comparing to terrestrial exercises, such as cycling [5], a high respiratory capacity compared to other athletes from different sports [6], and the higher energy cost in water than on land (e.g., running or cycling) due to the resistive force in the water (hydrodynamic resistance and drag) being larger than in the terrestrial environment (aerodynamic resistance) [7].

Swimming performance is also dependent on psychological, anatomical and physiological factors [8].

According to Crowley et al. [9], an increase in the strength development, power output, and neuromuscular function of a swimmer may improve their swimming performance [9]. The same authors reported that an increased level of muscle strength and power may largely affect swimming performance [9]. Therefore, COVID-19-induced home-based training may have a greater impact on swimmers than on land-based athletes.

## 2. Methods

A review was performed using the major relevant database search engines, PUBMED, MEDLINE, and GOOGLE SCHOLAR, using the specific terms, ‘COVID-19 AND swimming’, ‘COVID-19 AND sport’, ‘Swimmer training strategies’, and ‘detraining AND swimming’. Search terms were modified accordingly to fit the requirements or nuances of the databases used.

The term ‘COVID-19 and sport’ led to 1414 publications and the term ‘swimming nutrition’ to 1024 publications. When looking for ‘swimmer training strategies’, we obtained 76 publications, and obtained 46 publications with the term ‘detraining and swimming’. For ‘COVID-19 and swimming’, we obtained 18 publications, and, for ‘COVID-19 and swimming strategies’, there was only 1 recent publication; however, it was not relevant to our subject. The authors also screened the article titles and abstracts to determine eligibility. Case reports and field studies were examined for their clinical and practical relevance; animal and laboratory studies were not considered. Only studies with practical and clinical relevance were considered.

We organized this review starting with the current situation regarding COVID-19 in sports, and then we detail specific swimming training strategies to counteract the detrimental effects of the current COVID-19 home confinement.

## 3. COVID-19 and Water Environment

Currently, there is a lack of research concerning the transmission of viruses in swimming pools. To date, no transmission of COVID-19 in water environments has been proven.

According to the Centers for Disease Control [10], there is no “proof” that the microbe causing COVID-19 (i.e., SARS-Cov-2) can be spread to people through the water environment in pools, jacuzzi, healthy spas, or water play areas. Apparently, treatment with chlorine and other common disinfectants, such as bromine, ozone, or UV sanitizers, likely inactivates SARS-CoV-2 in the water [11]. Regardless, the virus can spread from person to person outside the water, or as children and adults play and relax at different areas, such as beaches and lakes. Therefore, the international swimming governing body FINA (Fédération Internationale de Natation) issued a document as a reference for the prevention of COVID-19 to its members, swimmers, and aquatic sport fans, advising to practice social distancing, protect oneself when training, not touch potentially contaminated surfaces, and be aware of good hand hygiene. This includes washing one’s hands regularly, using hand sanitizer or soap, and avoiding touching your face [12]. The quick spread of the pandemic around the world obliged FINA and national federations to cancel or postpone many national and international events.

## 4. Impact of COVID-19 on Major Swimming Events

On 11 March 2020, the World Health Organization (WHO) declared the presence of a new pandemic caused by this new virus called coronavirus (COVID-19), activating unprecedented postponement/cancellation of sporting events around the world, including the summer Olympic Games [13].

On 20 March 2021, a year after the start of the pandemic, the IOC and the International Paralympic Committee (IPC) were informed by the Japanese parties in the five-parties meeting about their conclusion to not allow foreign spectators at the Tokyo 2020 Olympic and Paralympic Games [14].

No other pandemic thus far has caused this many sporting event cancellations and as high a global impact as COVID-19 has. In this regard, FINA considered the impacts of the current situation with regard to the health of swimmers by assessing the risks of hosting forthcoming FINA competitions. Thus, a number of significant decisions were made to postpone or cancel major events; the most significant was the postponement of the most important event in a swimmer’s quadrennial preparation: the Tokyo 2020 Olympic Games. These decisions and the government policies to remain home pushed athletes to stop their daily activities, which resulted in the cessation of training.

## 5. The Effect of Swim Training Cessation

Starting March 2020, the majority of all sporting activities were ceased. Swimmers lost access to their main modality of training as they were no longer allowed to use swimming pools. Thus, swimmers were obliged to turn their homes into gyms and to be creative about their water-based training sessions (e.g., private pools and tethered swimming in their backyard), in order to maintain their fitness level and avoid excessive detraining. Detraining has been defined as the partial or complete loss of training-induced adaptations. Training cessation exerts negative effects on cardiovascular adaptation, muscle function, and energy metabolism [15]. Multiple factors, such as illness, injury, travel, or vacation, may disturb a swimmer’s training program, obliging them to adapt their physical activity schedule by reducing or even stop training [15]. The COVID-19 home confinement became another such factor.

According to a study by Ormsbee et al. [16], 35–42 days of swim cessation for healthy college-aged men and women but including easy light-physical exercise resulted in a (a) 1.3% increase in body weight; (b) 12.2% increase in body fat; (c) 7.7% decrease in peak oxygen consumption; (d) 7% decrease in the resting metabolic rate despite the preservation of lean mass; and (e) no difference in blood lipids or mood state. An early study by Costill [17] reported big changes in the metabolic characteristics in swimmers’ muscles following 1 to 4 weeks of training cessation. The respiratory capacity from the deltoid muscle (QO2) decreased by 50% following only 1 week of interruption, while the swimmers’ muscle glycogen content slowly reduced over the period of 4 weeks of training cessation [17]. Although muscular strength was not diminished over the same period, swimming power was significantly reduced following 4 weeks without training [17].

Impaired swimming performance was also associated with a decline in the kinematic variables (swim speed, stroke efficiency, stroke rate, and stroke index) following 4 weeks of training cessation [18]. Conversely, another study reported a surprising increase in swimming kinematics (swim speed, stroke efficiency, stroke rate, and stroke index), after 10 weeks of break in young swimmers [18,19]. These aforementioned findings could be explained by the puberty (growth and maturation) phase of the latter group of swimmers [18,19].

## 6. Impact of Different Training Strategies during Confinement

To counteract the detrimental effects of the current COVID-19 home confinement, swimmers around the world have been or are still trying to maintain their fitness level through different training methods.

### 6.1. Tethered Swimming

Tethered swimming is one potential strategy for swimmers to maintain their “feel for the water” and level of swimming (e.g., using a portable swimming pool in their backyard).

A recent investigation by Papoti et al. [20] on 21 age group swimmers reported no significant effect of 7 weeks of tethered swimming training compared to free-swimming. The experimental group had a high capacity of lactate production, suggesting that tethered swimming exercise in a training program may raise the anaerobic glycolysis contribution during exercise despite the lack of improvement observed in swimming force.

The absence of movement during tethered swimming can affect the outcome of this exercise and, thus, create mechanical difficulties for the swimmers, in comparison with normal swimming. Therefore, tethered swimming could affect the stroke movement [21]. However, changes in the stroke process do not affect the swim movement, particularly as the physiological responses are similar to in normal swimming [22]. Researchers suggested that this kind of exercise should be implemented with experienced swimmers in tethered swimming [21]. Otherwise, the results of inexperienced swimmers can be compromised [23].

Swimmers during the lockdown period must pay attention to the training load and intensity implemented with tethered swimming. In this period, it is recommended to focus on long sets at a moderate intensity with a short recovery time [24]. We are also aware that not every swimmer has the possibility of practicing tethered swimming at home. Other more accessible training tools/methods are described below.

### 6.2. Swimming Flume

A swimming flume provides a controlled environment for swimming training and permits the standardization of testing procedures and the evaluation of swimmers during swimming [25]. According to Hay and do Carmo [26], the swimming technique in a flume is different from pool swimming, and they found that swimmers used a higher stroke rate in a swimming flume than in a swimming pool at the same speeds. Conversely, a study by Xuhong Li et al. [27] on breaststroke swimmers reported that the stroke patterns and efficiency were similar for both a pool and swimming flume at corresponding speeds.

On the practical side, few swimmers have access to a swimming flume in their area or house backyard comparing to tethered swimming or other training tools/methods; however, according to a recent study [28], the force outcome of a flume swim at higher water-flow velocities is a better indicator of performance than tethered swimming exercise.

### 6.3. Swim Bench Training

The use of dry-land swimming ergometers was created to mirror the specific arm movements of all competitive swimming strokes (front-crawl, butterfly, breaststroke, and backstroke) [29]. Unfortunately, no cause—effect relationship was found between a swim bench and swimming performance [30]. Although the swim bench mimics the similar movements of the swimming strokes, it might not reproduce the same biomechanical demands in the water (e.g., the propulsive phase). Multiple researchers reported positive relationships between dry-land power on the swim bench and swimming performance [29,31]. However, these studies were correlational; they only described an association and not a cause–effect relationship [32].

Although swim bench training may not be an effective method to improve swimming performance [30], in the lockdown situation, swim bench training may be an efficient method to maintain the upper body adaptations of the swimmers by mirroring the swimming movements and may potentially improve a swimmer’s technical and strength deficiencies [33].

### 6.4. Rowing Ergometer

Rowing exercise is premised on the fact that the body is supported by a seat, which differs from most other types of exercise. In addition, both arms move at the same time. Rowing exercise is an endurance training activity [34]. It provides a non-impact, whole-body workout that translates well to almost any endurance sport. The motion also creates a strong core, which helps in swimming [35]. It is easy to obtain a challenging cardiovascular workout on a rowing ergometer. Rowing exercise can easily increase cardiovascular demands. During COVID-19 home isolation, a rowing ergometer can be an effective way to improve a swimmer’s fitness level in an indoor environment and also be used as a warm-up tool for a strength session [34]. However, an overload of rowing ergometer training may be associated with an increased injury risk [36].

### 6.5. Running and Cycling

Performing alternative training modes (i.e., running or cycling) may slow down detraining in athletes [37]. The integration of treadmill running and cycling exercise routines into swimming training schedules to raise the cardiovascular fitness level is also becoming more popular. The muscular adaptations influenced by land-based endurance training improve the aerobic processes and increase the maximum rate of oxygen consumption (VO2max) measured during incremental exercise, lactate threshold, and long-term endurance capacity. In contrast, swimmers and coaches favoring running and cycling exercise should keep in mind that these should be considered dissimilar mode cross-training options, as the contribution of leg kicking to the overall swimming speed has a partial contribution of only approximately 10–13%, whereas most of the propulsive force is generated by the upper body with approximately 87–90% [38,39].

A recent study by Currie et al. [40] reported that running might increase the volume of oxygenated blood pumped in the swimmer’s body. Therefore, running is beneficial for swimmers to raise their aerobic capacity and enhance their left ventricle function. Running is considered an alternative type of exercise to improve a swimmer’s cardiovascular system. On the other hand, according to Dintiman and Ward [41], cycling can offer a great alternative to injury reduction and rehabilitation. Cycling can also contribute to maintain the existing endurance level of the swimmers and, thus, maintain their subsequent performance.

### 6.6. Dry-Land Strength Training

Improvement of swimming performance is not only based on the sport-specific in-water training but also by other training alternatives, such as dry-land-strength training (i.e., strength and/or power training) [9]. Manning et al. [42], for instance, recommended to implement a specific dry-land training program to improve performance. According to multiple studies [29,31,43], a swimmer’s muscular strength can impact swimming performance, and dry-land strength training positively affected swimming speed in events ranging from a 50-m sprint [44] to a 400-m middle-distance event [32].

The propulsive phase in swimming (force and swim velocity) is mostly based on the upper-body musculature [38]; therefore, upper-body strength and power are imperative in swimming [29,31]. Additionally, lower-body strength and power are also key components to enhance start and turn performance in swimming, in addition to the initial swimming part of the race [45,46]. To have an indication of the swimming start performance, García-Ramos et al. [47] suggested to measure the vertical peak velocity during jumps. Beretić et al. [46] reported that the fastest swimmers in the first 10-m of a race were those who had the capacity to generate a large amount of force [46]. In addition, to have faster turns during races, swimmers must perform better in squat jump power, countermovement, and vertical jump height, and attain a faster speed at the push off phase of vertical jumping [48].

### 6.7. Circuit Training

Circuit training is often included in training routines to improve the cardiovascular fitness level, body composition, muscular strength, and endurance [49]. This kind of training is also recommended for the general preparation at the beginning of the season to improve aerobic fitness and to prepare young swimmers for strength training basics before moving to the gym [50].

Schmidt et al. [51] reported that high intensity short duration circuit training may improve muscle endurance. In another study, Paoli et al. [52] compared high-intensity exercise combined with endurance training and alone or low intensity endurance circuit training. The authors reported an improvement of body composition and blood lactate; thus, the high intensity exercise was more effective than the other regimes in improving performance or maintaining it.

Based on the above-mentioned information, circuit training could be an effective training strategy to be implemented within the program of swimmers during a period of home confinement, especially for novice swimmers [53].

### 6.8. Plyometrics

Several studies reported that plyometric training (PT) improved the explosive strength in men and women [54], pubescents, and adults [55] as well as in athletes from different sports.

The PT program is beneficial for swimmers because it does not significantly change the body composition and improve muscle power [56]. PT can improve the swimming start and turn performance. Practically, plyometrics are very effective because they do not require any special equipment and are not expensive [57].

According to Rebutini et al. [58], swimming starts can be improved by a specific PT program of long jumps. In another study, Bishop et al. [59] reported a beneficial effect of explosive power training through PT for a period of 8 weeks on swimming start performance. The aforementioned data suggest that plyometric training may be effective to enhance swimming performance in general and improve the start performance in particular. This type of training routine may also aid in kicking performance due to its large eccentric contribution [53].

### 6.9. Core Training

A training process is effective and beneficial when it includes an appropriate training of the abdominal muscles and torso [60]. The unique dynamic water environment of swimming training requires a good level of core strength and stability to better perform while overcoming the instability inherent to swimming [53]. Weston et al. [35] implemented a core training for a 12 weeks program in national level junior swimmers and found significant improvements in swimming performance.

According to these authors, swimming is based on the rotational axes and the control of this element requires stability in the lumbar and thoracic regions of the body. Therefore, the implementation of different exercises of core training allows for the increase in stability and better control of the swimming rotational axes while swimming. Controlling the body position while swimming and during the start and turns improves the swimmer’s performance, increases the stroke efficiency and reduces the distance traveled [61]. A recent study by Karpiński et al. [61] reported that the strengthening of the stabilizing muscles may be a beneficial addition to a swimmer’s training routine.

### 6.10. Eccentric Training

An alternative dry-land strength training method a swimmer could implement is eccentric overload training. This is defined as a type of strength training in which both the lifting and lowering phases of an exercise are performed but where load is added to the lowering (eccentric) phase. Consequently, the force produced is exceeded by the load applied to the muscle/muscle group, resulting in a high muscle force [53]. According to Chiu and Salem [62], PT has a better impact on different specialties’ athletes than traditional resistance training, especially because in improves the strength, power, and speed [62]. Therefore, including this type of training could potentially affect the strength of the swimmers and impact their performance after the home confinement period.

Eccentric overload training can be performed with flywheel inertial resistance equipment. Cuenca-Fernandez et al. [63] compared the effects of a traditional post-activation potentiation (PAP) stimulus (3 repetitions of lunge exercise) to a swim-specific PAP protocol (3 repetitions in a Yo-Yo squat flywheel device, modelling the start biomechanical position) on swimming start performance and compared both protocols to a control condition (swimming start following a traditional swimming warm-up). In conclusion, the authors reported that the swim specific PAP protocol using the squat flywheel device was more effective to improve both the first 5 and 15 m swim performance following a block start (5.7% and 2.4%, respectively), and this may be due to the similar movement pattern in both movements [63].

### 6.11. Instability Training

The nature of the water environment defines the specific type of training to be implemented by the swimmers in their training program. Instability training can mirror the unstable conditions of the swimmers in the water. Instable exercise conditions will force different group of muscles (synergistic, stabilizing, and antagonistic) of the swimmers to activate and coordinate [64]. Different degrees of instability can be attained through the use of various devices, such as wobble and rocker boards or foam rollers [53]. Unfortunately, there is a lack of studies regarding the effects of such training methods on swimming performance.

Endurance and strength training are very suitable training methods to maintain or enhance physical abilities in lockdown situations. A higher level of upper body power and muscular strength could affect the propulsive force in the water, the swimming biomechanics (e.g., stroke efficiency and stroke rate), and the swim velocity. On the other hand, an increase in lower body power and strength could affect the starts and turns during the race. Such improvements, along with technical skills and psychological preparation momentum may potentially help in the improvement of the swimming performance [53]. In this respect, it is important to keep in mind that strength training for swimmers in such particular periods will not replace the water training sessions of the swimmers but will attenuate the effect of in-water training cessation and aid in reducing the risk of overuse injury [65].

## 7. Additional Practical Recommendations for Swimmers

In addition to the traditional training methods, swimmers have the opportunity to strengthen other aspects of their preparation that may enhance their performance when resuming their normal training.

### 7.1. Yoga

Yoga is known to positively affect one’s mental health [66]. The combination of different elements, such as relaxation, meditation, and stretching, reduces stress and anxiety [66]. Following the current situation of the pandemic around the world, many swimmers have experienced great anxiety and stress. Inducing yoga into a swimmer’s daily routine may enhance their mood and balance the emotions and stress [67]. Incorporating yoga in a training routine may help a swimmer to control and be aware of their body and to improve their breathing coordination while also increasing overall strength [66]. These outcomes are subsequently transferred in the water particularly in that that the swimmer needs to be focused and aware of his body position and angle when swimming. The gained strength through various yoga poses is also transferable to the water and may contribute to the enhancement of the swimming efficiency and, thus, performance [67].

### 7.2. Respiratory Training

Competitive swimming requires the coordination between breathing patterns, buoyancy, and stroke efficiency [4] and this results in a higher tidal volume compared to terrestrial exercises, such as cycling [68].

One of the key elements of competitive swimming is the respiratory system; thus, swimming improves the inspiratory muscle function and consequently place extra load on the inspiratory dynamics [69]. This overload increases respiratory muscle fatigue and, in turn, impairs swimming endurance, performance, and breathing frequency [69]. It has also been shown to decrease the blood lactate concentration during exercise [70] and impair sympathetic activation.

30 min of resistive breathing training a day can enhance the pulmonary capacity, decrease airway resistance, and raise the volume of respiratory muscle endurance and the number of strokes per breath. This routine may contribute to a better oxygen diffusion and lower level of anxiety in competitive swimmers [71].

### 7.3. Flexibility

In competitive swimming, the shoulder is one of the most solicited groups of muscle, especially as the upper extremity contributes 90% of the propulsive force during swimming [72]. Therefore, swimming requires good shoulder mobility. A good flexibility program may also reduce injury risk [73]. Stretching is beneficial to develop and sustain the range of motion (ROM) and thus results in better stroke mechanics and stroke efficiency [74].

Static stretching results in the improvement of ROM and affects the flexibility of swimmers who require a large increase in their static ROM. However, it is not recommended to be performed before certain types of training, such as strength or explosive training. In swimming in particular, static stretching should be included in the training program as it is beneficial for their flexibility and may increase their ROM and musculotendinous compliance [75]. This type of training must be planned and appropriately incorporated in the overall program of the swimmer to achieve more flexibility and avoid the risk of injury [75].

### 7.4. Psychological Skills

Recently, interest in sport psychology techniques is growing rapidly because it is becoming one of the most important elements to improve competitive performance. Among these techniques, swimmers are using goal setting, imagery, self-talk, and arousal regulation because they are thought to enhance swimming competitive performance [76]. Simoes et al. [77] reported an improvement in swimming performance following the incorporation of a goal setting program for 9 swimmers using a multiple baseline design over 1 season, and a reduction in the swimming performance the following season when the intervention was ceased. The authors concluded that goal setting intervention pushed swimmers to devote more efforts towards their performance objectives and helped them commit to these goals [77].

Short et al. [78] reported that imagery impacted the ability of learning and performing as well as balancing the level of stress and anxiety and impacted cognitions, such as self-efficacy. They thus suggested that psychological skills training may be a useful intervention during and after the COVID-19 pandemic to help swimmers eliminate or avoid debilitating images and focus on their future goals (e.g., training, competitions, home isolation, and risk of contagion).

## 8. Nutrition for Confined Swimmers

The quarantine conditions and the special situation around the world following the current pandemic may have considerable risk factors regarding food consumption for swimmers. The anxiety and boredom also evoked by this situation may affect the quality of food consumed (poor quality) compared to standard living conditions [79]. The reduction in the amount of physical activity and the impaired nutritional habits could lead to a positive energy balance (i.e., weight gain) [80].

In these unprecedented times, even the most resilient swimmers must face serious challenges. Physical distancing, isolation, and disruptions in training and competition may negatively affect a swimmer’s body image and eating behaviors [81]. Cessation of regular, daily swim training may lead to weight gain, specifically fat mass, due to the short-term energy surplus, despite the smaller difference between energy intake and expenditure in the long term [16].

Swimming has been anecdotally associated with an increased appetite and risk of overeating compared with other sports activities. Therefore, swimmers and their coaches need to be aware of the negative outcomes of overeating during this COVID-19 pandemic situation and plan a nutritional routine accordingly. Swimmers should follow a specific schedule regarding their training frequency, intensity, duration and consider a healthy nutritional plan to maintain their cardiovascular fitness level, metabolic health, and a balanced body composition in this special period. These guidelines may induce training sessions of low volume/impact, high-intensity interval training one to two times per week and increase their cardiovascular fitness level by frequently consuming protein-rich, nutrient-dense foods [16,82].

## 9. Limitations and Future Research

The majority of the included studies examined the effect of training strategies on short term periods and the direct effects on subsequent activity. The COVID-19 pandemic created an unpredictable situation around the world in general, and specifically in sports. Athletes and coaches were unprepared for such a crisis; therefore, these recommendations would be stronger if they had been previously tested in the same situations with different protocols and with a longer period of study. Future studies on the minimal effective training dose to maintain physiological and performance adaptations in swimmers, as well as time-efficient retraining strategies are warranted.

## 10. Conclusions

The COVID-19 crisis and its consequences on the swimming community have created a myriad of challenges for swimmers around the world, including maintaining their fitness level and preparing to return optimally and safely to pool training and competitions. Unfortunately, the mental effects of the pandemic situation may impair a fast return to previous conditions [37]. The overall cancellation of different competitions and the loss of income have impacted swimmers. Consequently, elite swimmers must consider individual-level health monitoring during any pandemic period. The high levels of stress negatively affect the mood and, thus, reduce the ability of the body to resist infection [83].

In that regard, COVID-19-induced home-based training can help to avoid psychological pressures [82]. Moreover, season objectives should be adapted according to the current situation, and new ones implemented as soon as the training resumption and competition calendars are released. Finally, the above recommendations might help swimmers to maintain their fitness level and strengthen other skills in self-isolation as well as to learn to cope with the situation of quarantine and/or “physical distancing” in the case that such a pandemic situation is faced again in the future.
